# Local perfusion of capillaries reveals disrupted beta-amyloid homeostasis at the blood-brain barrier in Tg2576 murine Alzheimer’s model

**DOI:** 10.1186/s12987-023-00492-7

**Published:** 2023-11-22

**Authors:** Amira Sayed Hanafy, Alf Lamprecht, Dirk Dietrich

**Affiliations:** 1 Department of Neurosurgery, University Hospital Bonn, Bonn, Germany; 2 Department of Pharmaceutics, Institute of Pharmacy, University of Bonn, Bonn, Germany

**Keywords:** Blood-brain barrier, Alzheimer’s Disease, Tight junctions, ABC transporter, RAGE

## Abstract

**Background:**

Parenchymal accumulation of beta-amyloid (Aβ) characterizes Alzheimer’s disease (AD). Aβ homeostasis is maintained by two ATP-binding cassette (ABC) transporters (ABCC1 and ABCB1) mediating efflux, and the receptor for advanced glycation end products (RAGE) mediating influx across the blood-brain barrier (BBB). Altered transporter levels and disruption of tight junctions (TJ) were linked to AD. However, Aβ transport and the activity of ABCC1, ABCB1 and RAGE as well as the functionality of TJ in AD are unclear.

**Methods:**

ISMICAP, a BBB model involving microperfusion of capillaries, was used to assess BBB properties in acute cortical brain slices from Tg2576 mice compared to wild-type (WT) controls using two-photon microscopy. TJ integrity was tested by vascularly perfusing biocytin-tetramethylrhodamine (TMR) and quantifying its extravascular diffusion as well as the diffusion of FM1-43 from luminal to abluminal membranes of endothelial cells (ECs). To assess ABCC1 and ABCB1 activity, calcein-AM was perfused, which is converted to fluorescent calcein in ECs and gets actively extruded by both transporters. To probe which transporter is involved, probenecid or Elacridar were applied, individually or combined, to block ABCC1 and ABCB1, respectively. To assess RAGE activity, the binding of 5-FAM-tagged Aβ by ECs was quantified with or without applying FPS-ZM1, a RAGE antagonist.

**Results:**

In Tg2576 mouse brain, extravascular TMR was 1.8-fold that in WT mice, indicating increased paracellular leakage. FM1-43 staining of abluminal membranes in Tg2576 capillaries was 1.7-fold that in WT mice, indicating reduced TJ integrity in AD. While calcein was undetectable in WT mice, its accumulation was significant in Tg2576 mice, suggesting lower calcein extrusion in AD. Incubation with probenecid or Elacridar in WT mice resulted in a marked calcein accumulation, yet probenecid alone had no effect in Tg2576 mice, implying the absence of probenecid-sensitive ABC transporters. In WT mice, Aβ accumulated along the luminal membranes, which was undetectable after applying FPS-ZM1. In contrast, marginal Aβ fluorescence was observed in Tg2576 vessels, and FPS-ZM1 was without effect, suggesting reduced RAGE binding activity.

**Conclusions:**

Disrupted TJ integrity, reduced ABCC1 functionality and decreased RAGE binding were identified as BBB alterations in Tg2576 mice, with the latter finding challenging the current concepts. Our results suggest to manage AD by including modulation of TJ proteins and Aβ-RAGE binding.

**Supplementary Information:**

The online version contains supplementary material available at 10.1186/s12987-023-00492-7.

## Introduction

Alzheimer’s disease (AD) is a progressive neurological disorder distinguished by deterioration of memory and cognitive functions. Clinically, AD can be sporadic or familial, accounting for 95% and 5% of the cases, respectively [1]. Familial AD is predisposed by mutations often reported in amyloid precursor protein (*APP*), presenilin 1 (*PSEN1*) and presenilin 2 (*PSEN2*) genes [2], while sporadic AD develops in the elderly as a consequence of lifestyle and/or other concomitant diseases. The accumulation of parenchymal beta-amyloid (Aβ) plaques is considered the anatomical hallmark of AD brains, followed by intraneuronal neurofibrillary tangles. Under physiological conditions, the sequential cleavage of APP by β-secretase then γ-secretase produces Aβ protein [3]. The generated Aβ is released to the extracellular space and is shuttled across the blood-brain barrier (BBB) from *brain* to *blood* and vice versa by a group of receptors and carriers, which coordinate in order to maintain Aβ homeostasis and brain levels [3]. While the role of Aβ in physiological conditions is not fully understood, a recent study showed that the levels of soluble Aβ_1→42_ are associated with normal cognitive function and brain volume [4], emphasizing the importance of homeostatic control of Aβ levels in the brain.

The transporters involved in Aβ clearance to the blood stream include some ATP-binding cassette (ABC) transporters such as ABCB1 (P-glycoprotein) and ABCC1 (multidrug resistance protein-1) efflux pumps. While ABCB1 is located in the luminal membranes (facing the blood side), ABCC1 is found on both the luminal and abluminal membranes. Therefore, ABCB1 mediates the clearance of Aβ that was initiated by low-density lipoprotein receptor-related protein-1 (LRP1) on the abluminal vascular membranes [6]. Uptake of Aβ into brain parenchyma is mediated by the receptor for advanced glycation end products (RAGE) [7], while its efflux to the blood circulation is mediated by LRP1 [7]. However, it is noteworthy that some in vitro studies showed that LRP1 mediates the uptake of Aβ to the brain as well [8, 9]. Binding of advanced glycation end products and other ligands to RAGE induces NF-κB proinflammatory pathway and activates MAP kinases among other functions that are reviewed elsewhere [10]. It is noteworthy that Aβ is also produced in peripheral tissues including the liver, skeletal muscles and arteries [11]. Maintaining Aβ homeostasis in the brain relies on the equilibrium between Aβ influx and efflux across the BBB. Increased expression and/or activity of Aβ influx transporters or decreased expression and/or activity of Aβ efflux pumps could progressively lead to Aβ parenchymal accumulation, initiating a cascade of AD pathogenic events.

The BBB is formed by cerebral endothelial cells (ECs) interconnected by the protein complexes: tight junctions (TJ) and adherens junctions. The combined surface area of ECs ranges from 12 to 18 m^2^ in adult humans, rendering the BBB the largest tissue-blood exchange interface [12]. It has been increasingly evident that there is a robust correlation, and possible causation, between AD and the BBB integrity, which was reviewed elsewhere [13]. A number of AD-related changes of the BBB have been reported [14] including degeneration of ECs, reduced capillary length, serum leakage, altered level and activity of some BBB transporters, and disruption or misalignment of some TJ.

As the transport of Aβ across the BBB is a major event, many studies proposed the occurrence of endothelial injury in AD. In one study, it was found that Aβ_1→42_ oligomers induced the downregulation of some TJ proteins through RAGE-mediated mechanisms [15], while Aβ-RAGE interactions involving intracellular calcium mediated the disrupted expression of ZO-1, a TJ protein, in another study [16]. Moreover, it was found that incubating bEnd.3 cells with Aβ_1→42_ oligomers decreased the levels of three TJ scaffold proteins; claudin-5, occludin and ZO-1, and significantly upregulated RAGE protein levels [17]. It was also suggested that Aβ might get entrapped in the vascular walls during its clearance across the BBB [18] or that Aβ gets deposited in the periarterial interstitial fluid [19]. Collectively however, previous studies mainly assessed gene or protein expression rather than activity of transporters. Therefore, it is of great importance to functionally study Aβ efflux (via ABCB1 and ABCC1) and Aβ influx (via RAGE) at the BBB of AD brains. It is also essential to study the functionality of TJ in terms of limiting the paracellular diffusion (through the intercellular gap of ECs) and diffusion along the outer leaflet of endothelial membranes from the luminal to the abluminal side.

Experimental animal models have been developed to study several forms of AD [20]. One of these is the transgenic mouse line Tg2576 [21], which is considered a familial mouse model expressing the human Swedish *APP* mutation (B6;SJL-Tg(APPSWE)2576Kha). In the Tg2576 model, Aβ_1→42_ and Aβ_1→43_ levels are significantly higher than Aβ_1→40_ levels, which aligns with AD pathology in humans with *PSEN1* and *PSEN2* genetic mutations [21]. After 9 months of age, Aβ parenchymal and vascular deposits are evident and are accompanied by learning and memory impairments. Vascular Aβ deposits are associated with neighboring vessels, where the endothelium is lost, partially or completely, and the basal membrane undergoes splitting to accommodate the deposits [22]. After 12 months, all phenotype characteristics are manifested such as widespread Aβ plaques, gliosis and progressive cognitive impairment [21]. Therefore, we chose Tg2576 mice (14–18 months old) as an AD model to study functional alterations of the BBB and Aβ handling by ECs. After 15 months of age, it was reported that females develop 3 times more plaques than males, and that the levels of Aβ_1→40_ and Aβ_1→42_ are profoundly higher than in males [23]. To eliminate sex differences on cerebral amyloidopathy, we conducted the study exclusively in male mice.

The variation of the experimental approaches to study the BBB in relation to AD pathogenesis often leads to inconclusive, even contradictory, results [24]. Recently, we developed ISMICAP, a novel in situ microperfusion-based brain slice model [25]. It allows direct introduction of tracers and molecules of interest into cerebral vasculature in acute brain slices by micro-impalement of venules, while monitoring their subcellular interaction with ECs in real-time using high-resolution two-photon (2P) microscopic imaging. Acute brain slices are prepared immediately from freshly harvested brains. They are maintained in adequately oxygenated buffers and remain vital for a few hours. The slices are not further processed, for example in slice cultures. This procedure was developed in the 1950s by Henry McIlwain [26] to study metabolism and electrophysiological properties, and it has been adopted in many applications to this day. In this study, we applied ISMICAP to Tg2576 transgenic mice and obtained a detailed functional characterization of the BBB in the context of an AD mouse model. Compared to wild-type (WT) mice, we were able to identify significant BBB alterations in brain slices of Tg2576 mice.

## Methods

### Animals

Male transgenic Tg2576 (wt/tg) mice expressing the human Swedish APP mutation (*B6;SJL-Tg(APPSWE)2576Kha*) were purchased from Taconic Biosciences (Rensselaer, NY, USA). The mice were within 14–18 months old throughout all experiments. Age-matched WT Tg2576 (wt/wt) mice were obtained as a control. All experiments were conducted in accordance with the guidelines of the University of Bonn Medical Centre Animal-Care-Committee. According to the ARRIVE guidelines, all efforts were exhausted to minimize pain and suffering and to reduce the number of animals used. Animals were allowed water and food ad libitum, provided with nesting material and housed under alternating 12:12 h light/dark cycles (light-cycle 7am-7pm) at 22 ± 2 °C and 55 ± 10% humidity.

### Manufacturing of injection-pipettes

Custom-made pipettes were prepared as previously described [25]. Briefly, they were pulled from GB150F-8P glass capillaries using a P87 horizontal puller (Sutter Instruments). Then, the pipette tips were beveled at an angle of 45° using a 1300 M Micropipette Beveller (World Precision Instruments). Pipette tips were screened on a MF-900 microforge (Nashirigi) and only those with openings < 3 μm in diameter and debris-free were kept and used in experiments. After beveling, pipettes were kept for up to 4 weeks in air-tight containers.

### Preparation of acute brain slices

After anesthetizing with isofluorane inhalation, mice were decapitated, brains were collected and immediately submerged in ice-cold modified artificial cerebrospinal fluid (mACSF) containing (in mM) 87 NaCl, 2.5 KCl, 1.25 NaH_2_PO_4_, 7 MgCl_2_, 0.5 CaCl_2_, 25 NaHCO_3_, 25 glucose, and 75 sucrose (saturated with 95% O_2_/5% CO_2_). Horizontal cortical acute slices (300 μm) were obtained using a HM650 V vibratome (Thermo Fisher Scientific). The slices were kept in mACSF at 35 °C for 30 min then they were transferred to standard artificial cerebrospinal fluid (sACSF) containing (in mM) 124 NaCl, 3 KCl, 1.25 NaH_2_PO_4_, 2 MgCl_2_, 2 CaCl_2_, 26 NaHCO_3_, 10 glucose (saturated with 95% O_2_/5% CO_2_). Throughout the experiments, the slices were kept at room temperature in sACSF (saturated with 95% O_2_ / 5% CO_2_) at an equilibrium pH of 7.4. The slices were used for up to 6–8 h following their preparation.

### Experimental setup and intravascular perfusion

The ISMICAP experimental setup was constructed as previously detailed [25]. In brief, brain slices were placed in a submerged chamber, continuously perfused with aerated sACSF, of a 2P laser scanning microscope from Scientifica (2PIMS-PMT-20) or Bruker Scientific (Olympus BX51WI) equipped with a chameleon vision II laser (Coherent) and coupled with Scanimage r3.8 or Prairie View 5.4 software for scanning and acquisition, respectively. The wavelength, laser power and detector gain values in each experiment were optimized with respect to the fluorophore used. Within each slice, thin-walled venules (30–50 μm in diameter) were optically located and considered appropriate for impalement. Perfusion pipettes, containing 5 µl of the test fluorophore, were clamped to a micromanipulator (Luigs & Neumann) connected to a pressure pump (ALA Scientific Instruments). After puncturing the venular membrane with the pipette tip, a pressure of 100 mmHg was applied to flush the vasculature with the fluorophore solution and maintained throughout the experiment.

### Biocytin tetramethylrhodamine assay

Biocytin-tetramethylrhodamine (TMR, Sigma-Aldrich) was diluted in sACSF to 100 µM and perfused into cortical vessels. Z-stacks (plane distance 1 μm) of capillaries were recorded 5 and 30 min after starting pressure application at λ_ex_ 820 nm. The distance between the impalement point and the imaged capillaries was 100–120 μm. Spatial fluorescence profiles of regions of interest (ROIs) perpendicular to capillaries were determined using ImageJ [27] and normalized to the respective maximal intravascular fluorescence. To assess the integrity of TJ, the extravascular fluorescence intensities within 10 μm in a 3-µm distance of either side of capillaries were quantified at 5 and 30 min, normalized to the respective intravascular fluorescence and averaged. All measured intensities were background corrected.

### FM1-43 assay

FM1-43 (Thermo Fisher Scientific) at a concentration of 40 µM in sACSF was intravascularly perfused for 10 min. Z-stacks and single plane images of capillaries were acquired 2 and 10 min after pressure application at 840 nm. All imaged capillaries were within 50–100 μm distance from the impalement point. Spatial fluorescence profiles were determined as previously described. Fluorescence intensities within the luminal (lumen side) and abluminal (brain side) walls of ECs in capillaries were quantified at 10 min by manually outlining the ROIs. The abluminal fluorescence was normalized to the luminal one for the respective EC. All fluorescence values were background corrected.

### Calcein-AM uptake assay

Calcein-AM (Thermo Fisher Scientific) was prepared in sACSF to a working concentration of 20 µM and perfused intravascularly for 30 min. Calcein-AM, a non-fluorescent membrane-permeable molecule, is converted to fluorescent membrane-impermeable calcein by cytoplasmic esterases of ECs. As calcein-AM is non-fluorescent, TMR (100 µM) was added to the perfused solution to evaluate the impalement quality. Z-stacks were recorded at 0, 15 and 30 min at 920 nm. To achieve efflux protein inhibition, the slices were preincubated for 30 min with 1 µM Elacridar (ELA, Tocris Bioscience) or 0.6 mM probenecid (PRO, Tocris Bioscience) solutions in sACSF, individually or combined. During perfusion and imaging, the slices were constantly perfused with the respective inhibitor(s) solution. Dose-response experiments were conducted in slices preincubated with either ELA (0.2, 0.6 and 1 µM), PRO (0.2, 0.6 and 1 mM) or a mixture of both. To quantify calcein accumulation at 30 min, ROIs were carefully selected to contain the cytoplasm of ECs in capillaries, while the respective fluorescence intensities at 2 min were subtracted to eliminate inter-recording variations. All measurements were background corrected.

### Beta-amyloid uptake assay

Beta-amyloid (Aβ) monomers were prepared by dissolving fluorescent 5-FAM-Amyloid β-Protein (1-42) trifluoroacetate salt (Bachem) in ice-cold 1,1,1,3,3,3-Hexafluoro-2-propanol (HFIP) (Sigma-Aldrich) to the concentration of 100 µM [28]. The solution was incubated at room temperature for 1 h then on ice for 1 h and finally aliquoted. The excess HFIP was allowed to evaporate overnight and the aliquots were stored at − 80 °C. Before experiments, aliquots were reconstituted in DMSO (0.7% final DMSO concentration) and diluted in sACSF to 3.5 µM Aβ solution. To ensure that only Aβ monomers were perfused, the solution was kept on ice and filtered with 0.45 μm syringe filter directly before impalement. The Aβ solution was perfused intravascularly for 30 min, then flushed out with sACSF (for 30 s). To inhibit RAGE receptor, the slices were preincubated for 30 min in 10 µM FPS-ZM1 (Merck Chemicals GmbH). Aβ was flushed out with FPS-ZM1 (30 s) in experiments involving RAGE inhibition. Overlays of single-plane images of fluorescence and laser DIC scans were recorded at 810 nm. For comparability, the depth of all imaged capillaries and venules was kept between 20 and 40 μm, while the distance between the analyzed capillaries and impalement point was 50–100 μm. For fluorescence quantification in the luminal membranes of ECs, we used straight-line ROIs across vascular walls, which were carefully positioned not to include tissue autofluorescent aggregates. All intensities were background corrected.

### Data processing and statistical analysis

Unless otherwise specified, all images were raw data without denoising or smoothing. For better visualization, red, green, yellow and royal look-up tables (LUT) in ImageJ were used whenever relevant. Royal is a color map, coding low-intensity pixels ‘blue’ and high-intensity pixels ‘white’. Data are presented as mean ± standard error of the means (SEM). The number of analyzed ROIs (n_ROI_) was specified in the figure legends. According to the number of groups/variables, either one-way ANOVA, two-way ANOVA or two-tailed unpaired Student’s *t*-test were used. Pairwise multiple comparisons were corrected for using Tukey’s post *hoc* test (for two-way ANOVA) or Holm-Sidak post *hoc* test (for multiple *t*-tests). Differences were considered statistically significant at *P* < 0.05. All statistical tests applied were two-sided. Statistical analysis was performed using GraphPad Prism 9.0.2 (GraphPad Software, CA, USA).

## Results

### Tight junctions in Tg2576 cortical capillaries are considerably disrupted

We tested the integrity of TJ in the cortical capillaries of AD Tg2576 mouse brain. Normally, TJ restrict the passive diffusion of molecules through the intercellular cleft between adjacent ECs [25]. Hence, applying a fluorescent tracer intravascularly and quantifying its extracellular fluorescence in the vicinity of capillaries represent a read-out of the leakiness of TJ [25].

**Fig. 1:**
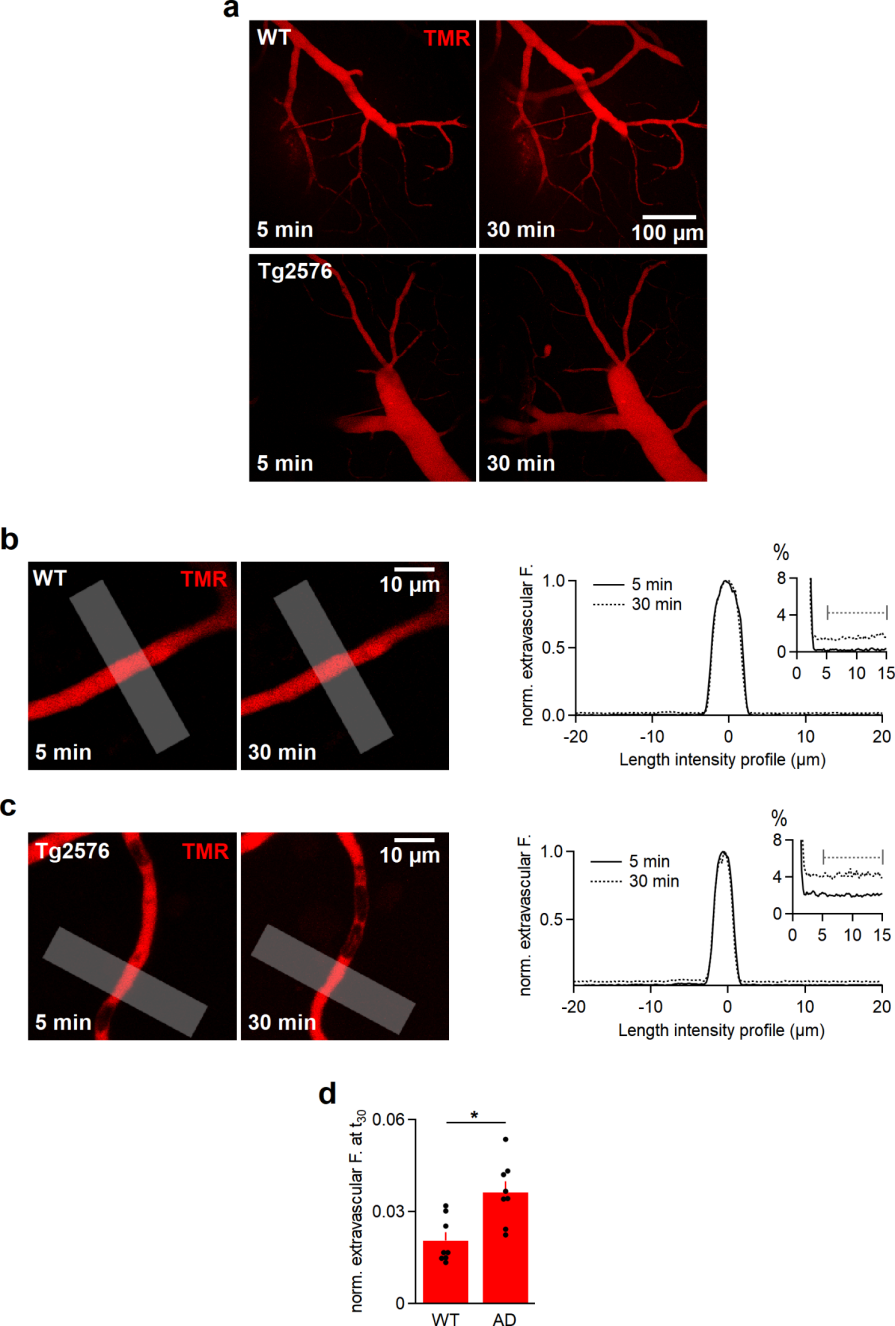
The extracellular diffusion of TMR is significantly increased in Tg2576 than wild-type mice. **a**, Maximum intensity projections (MIPs) of TMR-perfused vascular tree in the cortices of WT mice (top) and Tg2576 mice (bottom) mice 5 and 30 min after impalement. **b**, A single frame of a capillary in a wild-type cortical slice at 5 and 30 min (left) and the corresponding spatial fluorescence profiles (right) of a line profile normalized to the intravascular fluorescence. The analyzed line profiles (grey, 10-µm thick) were within 10 μm in a 3-µm distance to the sides. The extravascular fluorescence was 1.3 ± 0.03% at 30 min (the dashed bar region was averaged for quantification, see inset). **c**, A single frame of a capillary in Tg2576 cortex recorded at t_5_ and t_30_ (left) and the corresponding spatial fluorescence profiles (right) of a line profile. After 30 min, the extravascular fluorescence rose to 4.2 ± 0.04% (the dashed bar region was averaged for quantification, see inset). **d**, Quantification of extravascular fluorescence intensities within the specified ROIs across capillaries, 100–120 μm away from the impalement point, in WT and Tg2576 mice at 30 min. Data are represented as mean ± SEM. All intensities were background corrected and normalized to the intravascular fluorescence. The asterisk denotes a statistically significant difference (Student’s *t*-test, *P < 0.05). Data were obtained from 10 injections/4 animals (n_ROI_ WT mice = 8, n_ROI_ Tg2576 mice = 8)

A venule (30–50 μm in diameter) was located within the cortex and pierced with a glass pipette containing the fluorescent tracer, which was flushed into the vasculature via applying pressure on the back of the pipette [25]. Upon intravascular perfusion of TMR, rapid filling of the vascular system was observed in WT and Tg2576 mice within 5 min and the perfusion was continued for 30 min (Fig. 1a). We measured the extravascular fluorescence surrounding capillaries that were at least 100–120 μm distant from the impalement point (Fig. 1b,c). The intensity of extravascular fluorescence was quantified within a length of 10 μm in 3-µm distance of either side of capillaries at 5 and 30 min post perfusion. In WT mice, only 2.04 ± 0.3% (*n* = 8) of the intraluminal fluorescence was observed around capillaries at 30 min (Fig. 1b,d). In contrast, the extravascular fluorescence in Tg2576 mice within 5 min rose to 2.0 ± 0.3% (*n* = 8), and increased to 3.6 ± 0.3% after 30 min (Fig. 1c,d, significantly different from WT mice, Student’s *t*-test, *P* < 0.05).

**Fig. 2:**
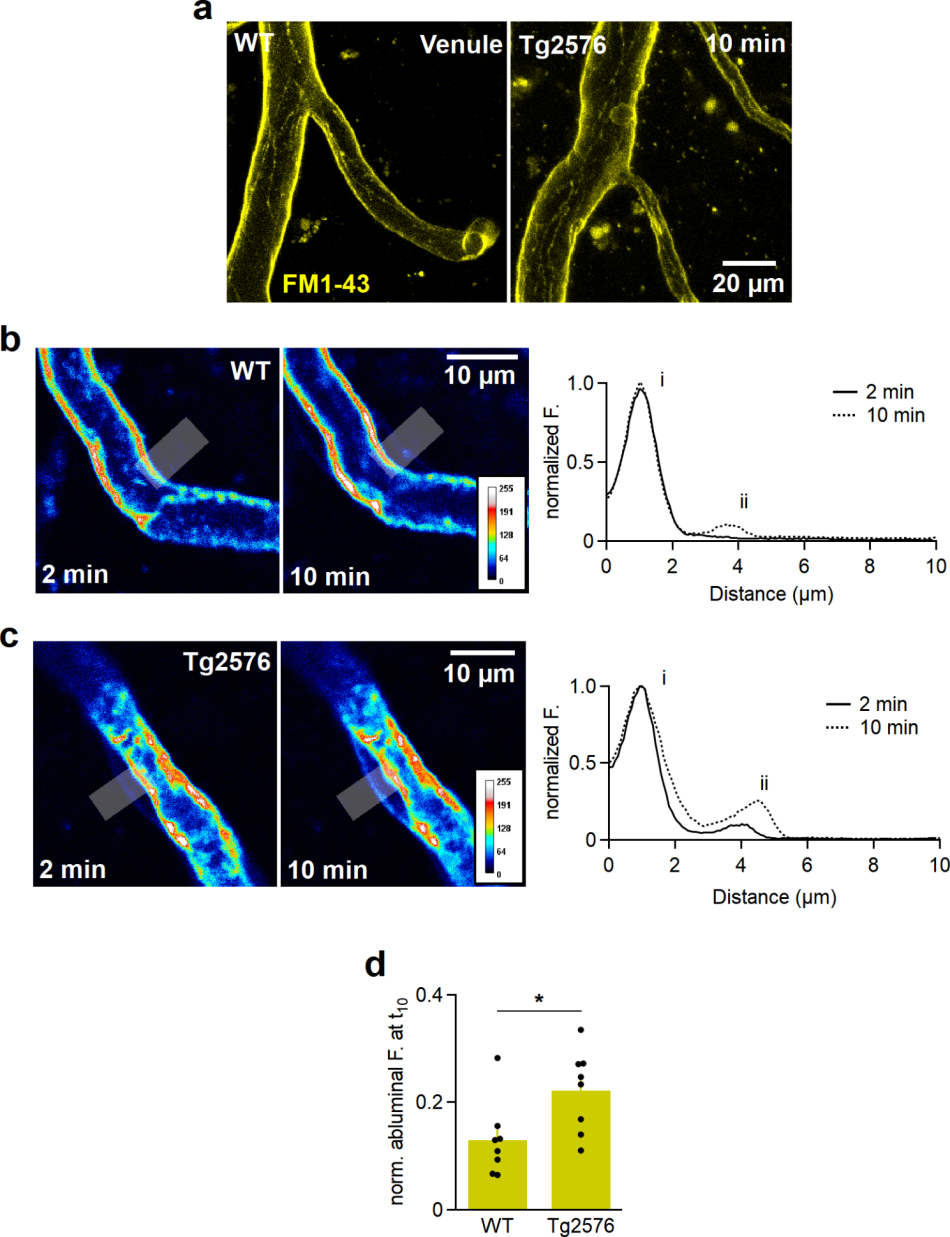
FM1-43 probe diffuses at a faster rate from the luminal to abluminal walls of endothelial cells in Tg2576 mouse brain tissue. **a**, MIPs of venules perfused with FM1-43 for 10 min in WT and Tg2576 mouse brains demonstrating the outlined ECs across the walls. **b**, A capillary in a WT cortical slice perfused with FM1-43 at 2 and 10 min (left) and the corresponding spatial fluorescence profiles (right) of a line profile (grey line, 5-µm thick) across the capillary wall normalized to the intravascular fluorescence. After 2 min, only the luminal membrane is stained (i), while the labeling of the abluminal membrane (ii) can be detected after 10 min. The LUT ‘royal’ is a color map that codes low-intensity pixels ‘blue’ and high-intensity pixels as ‘white’. **c**, A capillary in a Tg2576 cortical slice recorded at t_2_ and t_10_ (left) and the corresponding spatial fluorescence profiles (right) of a line profile normalized to the intravascular fluorescence. The labelling of the abluminal membrane can be detected at as early as 2 min after starting the FM1-43 perfusion, which intensifies over time (ii). **d**, Quantified fluorescence intensities within the abluminal membranes of capillaries in WT (n_ROI_=8) and Tg2576 (n_ROI_=8) mice. After 10 min, the diffused FM1-43 to the abluminal membranes in Tg2576 mice was 1.7-fold higher than in WT mice (Student’s *t*-test, *P < 0.05). The measured intensities were background-corrected and normalized to the corresponding luminal fluorescence intensities. Data are represented as mean ± SEM, obtained from 17 injections/5 animals

Next, we investigated the diffusion of FM1-43 styryl dye within the lipid membranes of ECs from the luminal side to the abluminal side. Typically, the proteins of TJ have extra- and intramembranous components. The intramembranous component is embedded into the lipid bilayers and undergoes protein-protein cis assembly via the interactions between transmembrane helical domains [29] and strongly limits the diffusion from luminal to abluminal compartments along the membrane [25]. The extramembranous component on the opposing membranes interact to fully seal the intercellular gaps, thus limiting paracellular diffusion. When applied to the lumen of the vascular system, FM1-43 initially labels the luminal membranes of ECs and reveals a reticular staining pattern outlining the membranes and borders of ECs [25], which could be clearly most detected in venules (Fig. 2a). FM1-43 only very slowly bypasses the TJ barrier and accumulates in abluminal membranes of ECs [25]. In the WT mice of the present study, which were much older than those used in our previous study (18-month-old *versus* 1-month-old), we also initially (2 min) observed only the staining of luminal membranes (Fig. 2b). After 10 min, a slight staining of the abluminal membrane of ECs was visible (Fig. 2b). In contrast, the staining of the abluminal membrane in Tg2576 mice already appeared after 2 min (Fig. 2c**)** and reached significantly higher levels of 0.13 ± 0.02 and 0.22 ± 0.03 after 10 min in WT and Tg2576 mice, respectively (Fig. 2c,d, *n* = 8 each group, Student’s *t*-test, *P* < 0.05). This apparently accelerated diffusion of FM1-43 from the luminal to abluminal sides indicates the reduced integrity of the intramembranous component of TJ [25].

### Functionality of ABC efflux transporters is altered in Tg2576 brains

**Fig. 3:**
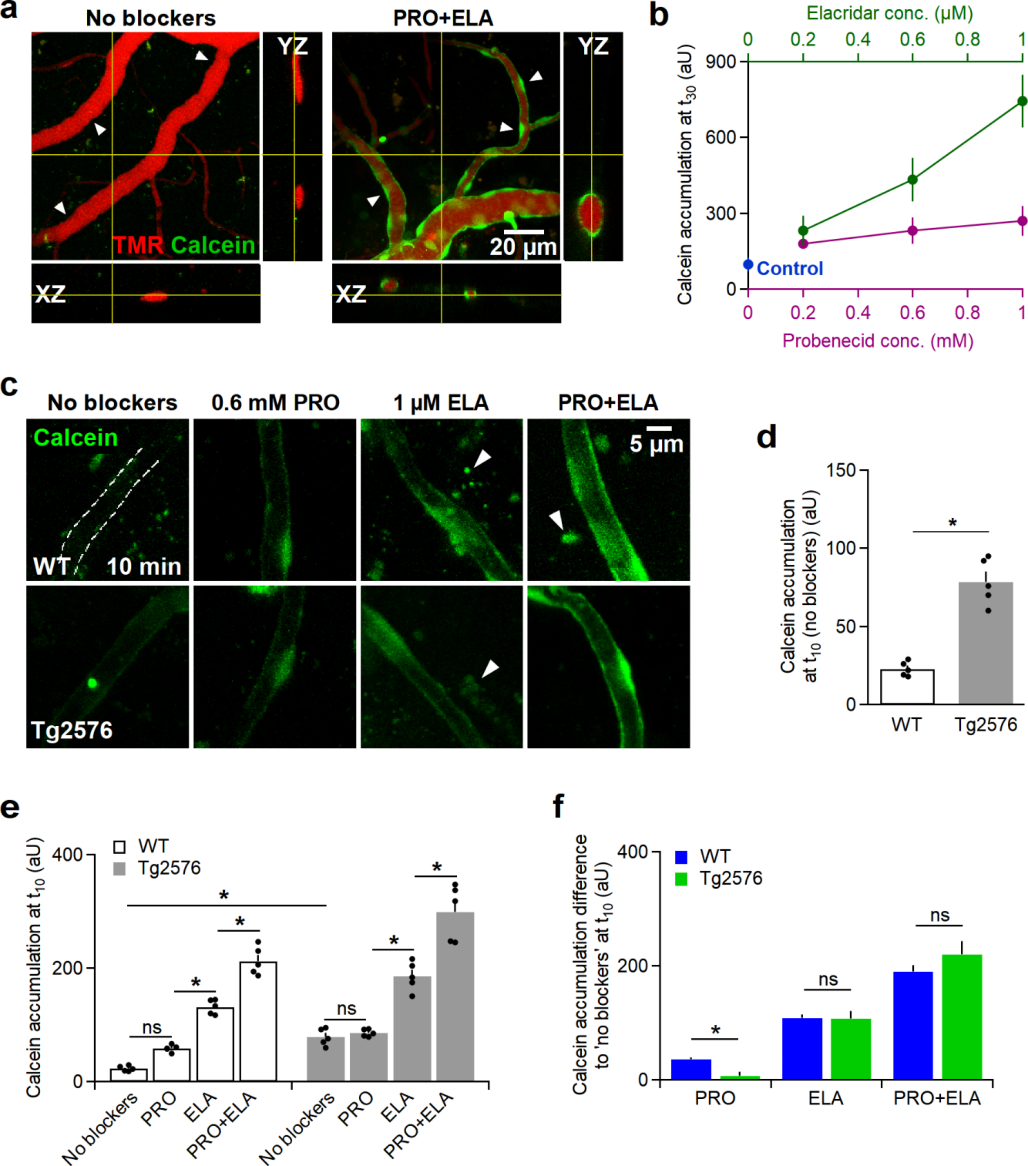
The endothelial efflux systems in Tg2576 mice are significantly hindered. **a**, Cross-sectional views of cortical vessels perfused with calcein-AM for 10 min under control conditions (left) and simultaneous inhibition of ABCC1 and ABCB1 transporters with 0.6 mM PRO and 1 µM ELA, respectively (right). Without blockers, no calcein accumulation was detected in endothelial cells (arrow heads, left), while it was observed after applying PEO and ELA (arrow heads, right). **b**, Dose-response curve showing the concentration-dependent accumulation of calcein 30 min after applying PRO or ELA. The ‘control’ datapoint denotes calcein accumulation in absence of both blockers. **c**, MIPs of capillaries recorded at 10 min demonstrating calcein accumulation under control conditions as well as inhibition of ABCC1, ABCB1 or both by 0.6 mM PRO, 1 µM ELA or a mixture of both, respectively, in WT mice (top row) and Tg2576 mice (bottom row). In WT mice capillaries, no calcein accumulation could be observed under control conditions, therefore the capillary’s lumen was manually outlined (dashed ROI) using simultaneously perfused TMR. Arrowheads denote extravascular autofluorescent aggregates in aged mice. **d**, Calcein accumulation in ECs at 10 min in absence of blockers in Tg2576 mice was significantly higher than that in WT mice (n_ROI_ WT mice = 5, n_ROI_ Tg2576 mice = 5, *P < 0.05). **e**, Quantification of calcein accumulating at 10 min in WT and Tg2576 mice in absence and presence of ABCC1 and ABCB1 blockers. A similar pattern of calcein accumulation was observed within both WT and Tg2576 mice groups. Compared to control conditions, applying PRO led to insignificant calcein accumulation, while applying ELA or ELA + PRO mixture resulted in significantly increased calcein accumulation. Data, presented as mean ± SEM, were obtained from 24 injections/6 animals (n_ROI_ = 5 for all groups, two-way ANOVA, *P < 0.05, ns; insignificant difference, see supplementary information for details). **f**, Inhibitor-induced increases in calcein fluorescence with respect to control (no blockers) conditions. PRO-sensitive ABC transporters were almost absent in Tg2576 mouse brains, while ELA-sensitive extrusion remained unchanged (multiple *t*-tests, *P < 0.05, ns; insignificant difference).

The activity of two ABC efflux transporters, ABCB1 (P-glycoprotein) and ABCC1 (multidrug resistance protein-1), was assessed. Normally, calcein-AM does not accumulate in ECs due to the active extrusion by efflux pumps. While calcein is a substrate for ABCB1, both calcein and calcein-AM are substrates for ABCC1 [30]. Calcein accumulation can only be detected in the ECs lining vascular walls after blocking ABCB1 or ABCC1 by ELA and PRO, respectively (Fig. 3a). The accumulation of fluorescent calcein increased in a concentration-dependent manner with ELA and PRO (Fig. 3b). The concentration dependence was consistent with the reported IC50 values of ELA (0.19 µM) [31] and PRO (0.5–0.8 mM) [32] for ABCB1 and ABCC1, respectively, and supported the importance of these transporters in removing calcein from ECs under control conditions. Under control conditions (absence of inhibitors) in WT mice, calcein was almost undetectable in ECs after 10 min as observed previously [25]. In contrast, we observed a moderate but significantly higher accumulation of calcein after 10 min perfusion in Tg2576 mice; suggesting that AD mice show a lower basal activity of calcein extrusion compared to WT mice (Fig. 3c,d).

We employed the inhibitors PRO and ELA at submaximal blocking concentration of 0.6 mM and 1 µM, respectively, to probe which type of ABC transporter may be affected. When adding either PRO or ELA to the WT group, we observed a marked and strong increase in the accumulation of calcein fluorescence in ECs (Fig. 3e, see supplementary information). In contrast, PRO had almost no effect in Tg2576 mice but ELA led to a significant accumulation of fluorescence in ECs (Fig. 3c,e, two-way ANOVA, *P* < 0.05). This inhibitor-induced increase in fluorescence is proportional to the number of calcein molecules trapped in ECs and, conversely, a measure of the number of calcein molecules which were extruded by the ABC transporter before blocker application per 10 min. Thereby, as a first approximation, this increase is proportional to the reduction in calcein efflux rate caused by the transporter inhibitors.

In order to reveal the effect of genotype on calcein accumulation, per treatment, the mean calcein accumulation under ‘no blocker’ condition was subtracted from each of the mean values corresponding to each blocker treatment (within the same genotype, Fig. 3f). To estimate the reliability of those new mean values, we used rules of error propagation to calculate the new SEM values (which are plotted along with the mean values, Fig. 3f). A direct statistical comparison of mean values based on their SEM was performed using a multiple *t*-tests approach followed by Holm-Sidak post *hoc* test. This analysis clearly showed that PRO-sensitive ABC transporters were almost absent in Tg2576 mouse brain tissue (*P* < 0.05), while ELA-sensitive extrusion was unchanged. We noticed in WT mice that the combined application of PRO and ELA increased calcein fluorescence in ECs more than the sum of the elevations in fluorescence levels observed when applying them individually. This supra-additive effect could be explained by the higher calcein levels reached upon combined inhibitor application (Fig. 3c,d). If calcein reaches higher levels within ECs, for instance by applying ELA, a larger fraction of intracellular binding sites of ABC transporters, in particular PRO-sensitive binding sites, will be bound by calcein which will result in an elevated efflux rate of PRO-sensitive transporters. The additional application of PRO will then lead to an enhanced reduction in efflux rate and a larger increase in fluorescence than observed if PRO was applied under baseline conditions (i.e. without ELA).

We observed an even more pronounced supra-additive effect upon combined application of PRO and ELA in the Tg2576 group (Fig. 3f). This was unexpected as PRO was ineffective under baseline conditions, suggesting that ECs in the AD group may lack PRO-sensitive transporters. The supra-additive effect of combined inhibitor application may imply that PRO-sensitive extrusion mechanisms are present but with altered properties in the AD group. For instance, a strong decrease in their affinity for calcein may render them ineffective for extrusion under baseline conditions when calcein concentrations in ECs are low, and let them noticeably participate in calcein extrusion only when calcein levels are strongly elevated by ELA application.

### Reduced binding of beta-amyloid to luminal membranes of endothelial cells in Alzheimer’s Tg2576 brains

To assess the potential uptake of Aβ monomers by ECs, we perfused the vasculature with fluorescently tagged Aβ solution (3.5 μm, 5-FAM-tagged) for 30 min, then briefly with sACSF (for 30 s) for flushing to reveal fluorescent Aβ interacting with ECs by 2P imaging. Aβ monomers bind to RAGE, which is expressed in the luminal membranes of ECs.

**Fig. 4:**
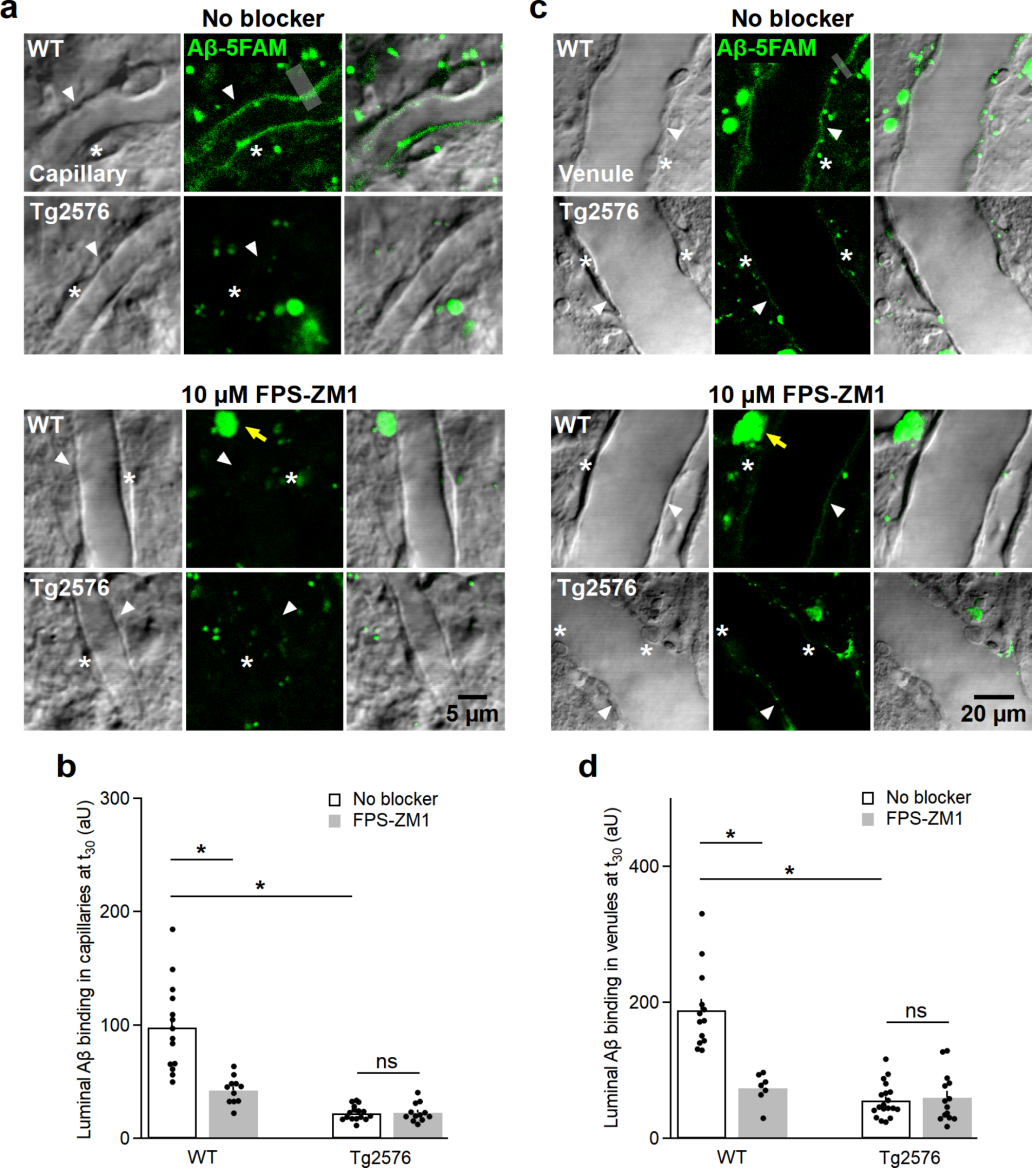
Beta-amyloid uptake in capillaries and venules of Tg2576 mice is significantly decreased. **a**, Single frames of capillaries 30 min after perfusing 5-FAM-tagged Aβ monomers in WT and Tg2576 murine vasculature with/without inhibiting RAGE transporter by 10 µM FPS-ZM1. All images are displayed in parallel as fluorescence scan, laser DIC scan and an overlay of both. In WT mice, Aβ fluorescence is localized at the luminal walls (arrow heads) under control conditions, which significantly decreased after RAGE inhibition by FPS-ZM1 (see **b**). In Tg2576 mice, negligible Aβ uptake was noted under both experimental conditions (see **b**). An example of line profiles used for analysis is shown (grey line, 3-µm thick). Asterisks signify ECs, while yellow arrows refer to autofluorescence commonly found in aged tissue. **b**, Quantification of luminal fluorescence intensities in capillaries under control conditions (n_ROI_ WT mice = 14, n_ROI_ Tg2576 mice = 16) and RAGE inhibition (n_ROI_ WT mice = 11, n_ROI_ Tg2576 mice = 12) represented as mean ± SEM (two-way ANOVA, *P < 0.05; ns, insignificant, see supplementary information). **c**, Single frames of venules after perfusing 5-FAM-tagged Aβ monomers for 30 min under control conditions and after preincubation with 10 µM FPS-ZM1 in WT and Tg2576 murine vasculature. In WT mouse brain tissue under no-blocking condition, Aβ fluorescence is localized within the luminal walls (arrow heads), which significantly decreased upon inhibiting RAGE with FPS-ZM1 (see d). Significantly lower Aβ uptake was recorded in Tg2576 venules, which remained unaltered after applying FPS-ZM1 to block RAGE (see d). An example of the analyzed line profile is indicated (grey line, 3-µm thick). Similarly, ECs are marked by asterisks, while yellow arrows indicate autofluorescence. **d**, Quantification of luminal fluorescence in venules under no-blocking condition (n_ROI_ WT mice = 13, n_ROI_ Tg2576 mice = 19) and RAGE inhibition (n_ROI_ WT mice = 7, n_ROI_ Tg2576 mice = 14) represented as mean ± SEM. (two-way ANOVA, *P < 0.05; ns, insignificant, see supplementary information). Data were collected from 10 injections/4 animals

To identify ECs, we scanned the fluorescence along with a transmitted light (laser) DIC channel revealing the vascular cells in the acute brain slice (Fig. 4a,c). In WT mice, we observed a thin line of green fluorescence along the luminal membranes of capillary ECs (Fig. 4a), while the cytoplasm and abluminal cell membranes of ECs were not stained. It is noteworthy that the luminal and abluminal membranes can be distinguished only at the cell bodies of ECs (i.e. where the nucleus is located), as they nucleus separates the two membranes by several micrometers. Therefore, it is concluded that Aβ monomers were bound solely to the luminal membranes. The luminal line of fluorescence was significantly weakened when the slices were incubated and perfused with FPS-ZM1 (10 µM) (Fig. 4a,b). Line profiles were drawn across ECs to quantify luminal fluorescence intensities, which were background corrected using the parenchymal fluorescence value across the respective line profile.

FPS-ZM1 blocks the binding of Aβ to the V-domain of RAGE (*K*_*i*_ = 25 nM) [33]. Similarly, 5-FAM-tagged Aβ bound to ECs lining venules and this binding was significantly antagonized by FPS-ZM1 as well (Fig. 4c,d, two-way ANOVA, *P* < 0.05, see supplementary information). In Tg2576 capillaries and venules, we did not observe Aβ fluorescence of the luminal membranes of ECs and FPS-ZM1 was without effect (Fig. 4b,d, two-way ANOVA, *P* < 0.05, see supplementary information), suggesting that Aβ does not, or only minimally, bind to ECs or RAGE in Tg2576 mice.

Note that fluorescent parenchymal aggregates were recorded in all acquisitions even before 5-FAM-tagged Aβ application (*asterisks*, Fig. 4a,c), and likely represent autofluorescence in cells containing lipofuscin accumulation naturally occurring with ageing [34].

## Discussion

**Fig. 5:**
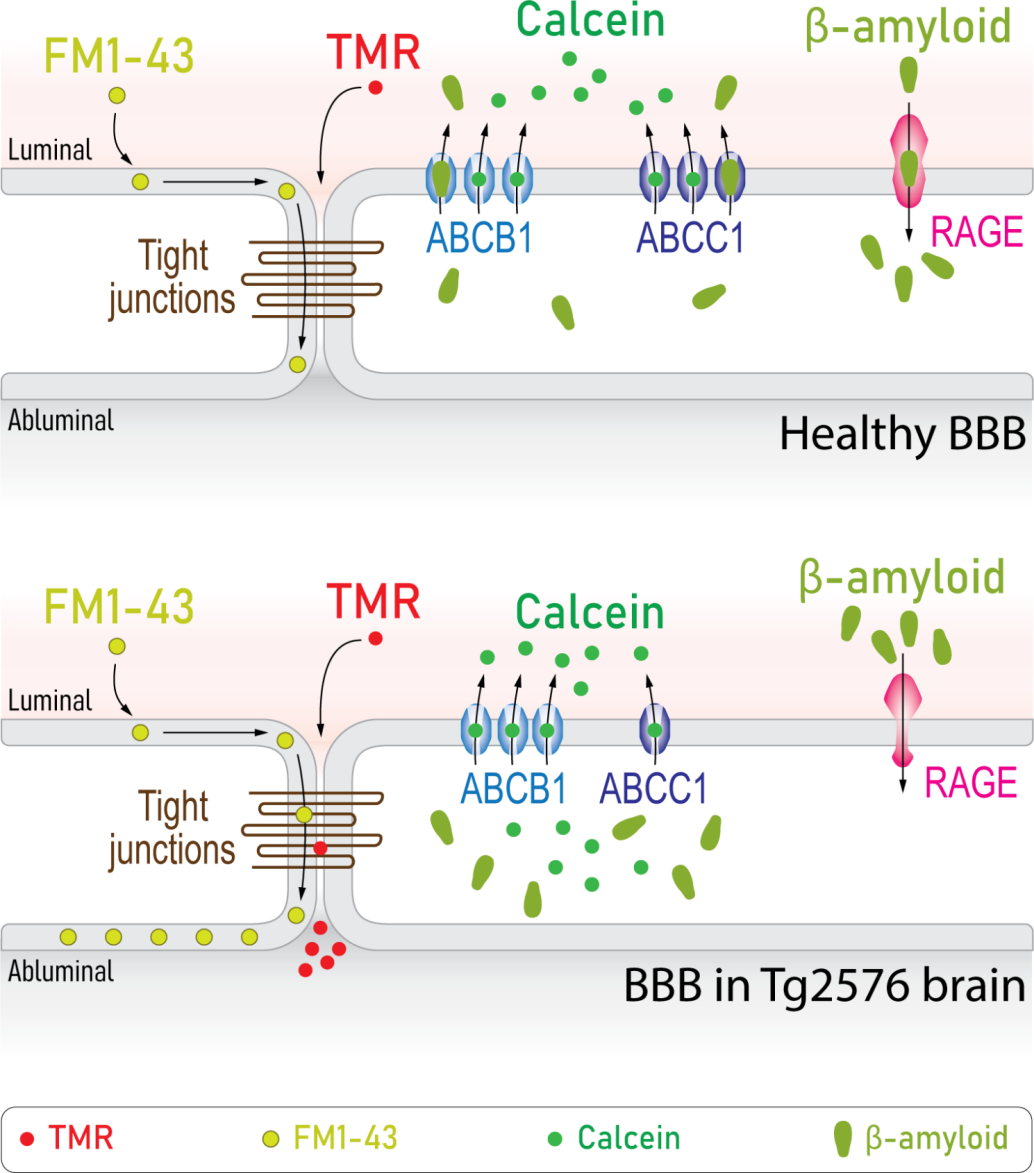
Schematic representation of the healthy blood-brain barrier (top) compared to pathological blood-brain barrier alterations identified in Alzheimer’s Tg2576 mouse brain (bottom) in the current study along with the associated experimental observations

In this study, we characterized the BBB in Tg2576 AD mouse line in comparison with age-matched controls. Our findings demonstrated a significant disruption of TJ as evidenced by increased TMR leakage to parenchyma and faster FM1-43 diffusion from the luminal to abluminal endothelial membranes (Fig. 5). A previous study showed parenchymal extravasation of Texas red-labelled bovine serum albumin after systemic administration in Tg2576 mice [35] even before forming senile plaques, while the BBB permeability was unchanged in *APP/PS1* mice [36] and *3xTg-AD* mice [37]. Expression studies in different in vitro and in vivo models showed that some TJ proteins are downregulated in AD including claudin-5, occludin and ZO-1, as reviewed elsewhere [38]. The conflicting results of BBB permeability in AD could be resulting from the used model, age group, brain region and/or experimental tracer. For example, it is commonly believed that the BBB is disrupted in epilepsy [39]. However, hippocampal tissue from chronic epileptic humans and status epilepticus mouse model exhibited a well-maintained BBB permeability to two differently sized tracers, TMR and Alexa488 labelled-bovine serum albumin, compared to WT samples when assessed with ISMICAP [25].

ABCC1 and ABCB1 are involved in regulating Aβ levels in the brain. Aβ_1→42_ and Aβ_1→40_ are substrates for ABCB1 [40], which plays a pivotal role in Aβ clearance. ABCB1 and LRP1 orchestrate Aβ clearance to the blood stream, where LRP1 mediates the uptake of Aβ from the parenchyma to the abluminal membranes [5]. Decreased ABCB1 expression was previously reported as a common AD manifestation [41] and restoring its function in isolated capillaries of Tg2576 brains in fact reduced Aβ levels [42]. ABCC1 is localized on both the luminal and abluminal membranes of ECs and it is involved in Aβ clearance as well [43]. In ABCC1 knockout mice, Aβ_1→42_ and Aβ_1→40_ levels were 12-fold and 14-fold higher than in control mice, respectively [43]. However, little is known about how ABCC1 is involved in AD pathogenesis. Here we show, for the first time, that the efflux activity of ABCC1 transporter is remarkably altered in Tg2576 brain tissue, as demonstrated by the significant calcein accumulation in ECs without applying any ABC blockers (Fig. 5), while the activity of ABCB1 transporter was unchanged. Interestingly, calcein accumulation in ECs of Tg2576 brain when ABCB1 and ABCC1 were blocked was higher than the sum of their individual inhibition. This supra-additive effect might be resulting from altered ABCC1 properties or lack thereof. It is also suggested that when endothelial calcein accumulation is low under control condition, there is little binding of calcein to ABCC1. Another potential explanation is that ABCC1 is localized in a submembranous compartment and gets rapidly recruited to the plasma membrane, similar to the delivery of glutamate receptors in neurons, only when calcein levels increase/other transporters are recruited.

RAGE is a multi-ligand receptor that plays significant roles in maintaining the equilibrium of Aβ. It has been reported that RAGE levels increase in brain endothelium leading to Aβ deposition in AD patients [6, 44]. FPS-ZM1 antagonizes RAGE in brain endothelium, decreasing the influx of circulating Aβ_1→42_ and Aβ_1→40_ in Tg2576 mice [33]. Perfusing fluorescent-tagged Aβ in WT mice resulted in their binding to the luminal membranes of ECs, while the cytoplasm and abluminal membranes remained unstained. The reason could be that Aβ uptake into the cytoplasm is too slow to be recorded within the experimental time (30 min) or that strong Aβ extrusion mechanisms at the abluminal membranes exist. In Tg2576 mice, the luminal binding of Aβ monomers was significantly lower than that in WT mice (Fig. 5). This finding may suggest a potential decline of RAGE activity in Tg2576 brain, as evident by the lack of inhibitory effect of FPS-ZM1 on the binding of Aβ to RAGE. However, it does not entirely negate that RAGE could be overly expressed. For example, it is possible that RAGE could be saturated by endogenous Aβ in Tg2576 brain tissue, which could also explain why RGAE inhibition had no effect on tagged-Aβ luminal binding. It could be that the binding of Aβ to RAGE is extended and decreases only minimally over several hours. It is suggested that the binding sites of RAGE are either less frequent or irreversibly blocked by endogenous Aβ. In a previous study, the in vivo uptake of circulating radiolabeled Aβ was quantified by homogenizing the entire hemisphere [45]. Given that Aβ binding to RAGE is reversible, this technique does not differentiate between unidirectional and bidirectional transport. As the whole brain tissue is homogenized, it is not possible to define whether the assayed Aβ is localized within microvessels, transcytosed into the parenchyma, endocytosed by ECs or only bound to the luminal membranes.

It is noteworthy that there is an interplay between TJ disruption, decreased ABCC1 activity and altered RAGE activity in Tg2576 mice. Disruption of TJ proteins was linked to Aβ accumulation via different mediating mechanisms. Using an in vitro murine endothelial bEnd.3 model, Aβ_1→42_ oligomers induced the downregulation of occludin, claudin-5 and ZO-1 via RAGE-mediated autophagy as evidenced by the increased LC3-II/β-actin ratio and decreased p-mTOR/mTOR ratio [15]. In another study, ZO-1 expression was disrupted in bEnd.3 cells via Aβ-RAGE interactions involving increased intracellular calcium levels [16]. In the *5xFAD* AD animal model, it was found that the capillaries near Aβ deposits in the brain parenchyma appeared disrupted or cut [16]. In human postmortem cortical tissue, the capillaries adjacent to Aβ parenchymal deposits exhibited a remarkable loss of occludin, claudin-5 and ZO-1 associated with clusters of NOX-2-positive microglia [46]. Therefore, multi-targeted therapeutic approaches to manage AD could have superior potentials over those targeting a single pathogenetic pathway.

Disease-modifying therapeutics for AD management have been primarily focusing on Aβ, especially in phase II and phase III clinical trials [47]. So far, only two disease-modifying treatments have been granted accelerated FDA approvals, which are the monoclonal antibodies aducanumab [48] and lecanemab [49]. Aducanumab reduces Aβ levels by binding to Aβ aggregates [50], while lecanemab binds to soluble Aβ photofibrils in early AD [51]. Our findings, coupled with ISMICAP potentials, identified alternative innovative therapeutic strategies. One potential therapeutic pathway could be to correct for the compromised integrity of TJ in AD by enhancing the expression of TJ proteins using gene therapy or tissue engineering. For instance, hippocampal transplantation of endothelial progenitor cells in an *APP/PS1* mouse model upregulated several TJ proteins, namely claudin-5, ZO-1 and occludin, which significantly enhanced cognitive and memory functions [52]. Interestingly, suppression of claudin-5 and occludin via siRNAs lowered Aβ_1→40_ levels in the brain of Tg2576 mice in another study [53]. How TJ proteins should be modulated to manage AD is yet to be closely investigated. Despite the extensive research on TJ modulators, they should be cautiously approached. To this day, new ways by which TJ proteins interact are being discovered. It has been increasingly evident that TJ are highly dynamic and adaptive to different pathophysiological demands. Interfering with how ‘tight’ the TJ are in AD context, while being potentially effective in Aβ clearance, may result in disturbing the homeostasis of other molecules, posing unexpected outcomes. In addition, the duration of action of TJ modulators should be considered in order to avoid complications due to sustained and/or uncontrolled TJ opening. For instance, the knockdown of claudin-5 in mice for 2 weeks resulted in lethal neuroinflammation [54]. Moreover, a previously examined therapeutic pathway was inducing the overexpression of ABCB1, which promoted Aβ clearance in Tg2576 AD model [42]. However, here we identified a decreased activity of ABCC1, while that of ABCB1 was unaltered in Tg2576 mice. Therefore, it is suggested that restoring ABCC1 functionality could be therapeutically promising, which has not been investigated yet to our knowledge.

It has been reported that inhibiting RAGE helps to slow down Aβ accumulation and decreases cognitive deterioration. Azeliragon, a small orally bioavailable molecule, was investigated as RAGE inhibitor, where it was found to lower total Aβ brain concentration and improve cognitive functions [55]. However, phase III clinical trial investigating Azeliragon (NCT02080364) was recently terminated because of lack of efficacy. In this study, we show that inhibiting RAGE was ineffective in lowering the luminal Aβ binding, which could be attributed to RAGE saturation by endogenous Aβ in Tg2576 mouse brain. Dissociating endogenous Aβ from RAGE could have therapeutic potentials and help to normalize Aβ homeostasis in AD brain tissue.

### Electronic supplementary material

Below is the link to the electronic supplementary material.

Supplementary Material 1

## Data Availability

The analyzed datasets in the current study are available from the corresponding author on reasonable request.

## Abbreviation

2P: Two-photon
ABC: ATP-binding cassette
AD: Alzheimer’s disease
APP: Amyloid precursor protein
Aβ: Beta-amyloid
BBB: Blood-brain barrier
DMSO: Dimethyl sulfoxide
ECs: Endothelial cells
ELA: Elacridar
HFIP: 1,1,1,3,3,3-Hexafluoro-2-propanol
LRP1: Low-density lipoprotein receptor-related protein-1
LUT: Look-up table
mACSF: Modified artificial cerebrospinal fluid
PRO: Probenecid
PSEN1: Presenilin 1
PSEN2: Presenilin 2
RAGE: Receptor for advanced glycation end products
ROIs: Regions of interest
sACSF: Standard artificial cerebrospinal fluid
SEM: Standard error of the means
TJ: Tight junctions
TMR: Biocytin-tetramethylrhodamine
WT: Wild-type

## References

[1] Liu P-P, Xie Y, Meng X-Y, Kang J-S (2019). History and progress of hypotheses and clinical trials for Alzheimer’s Disease. Signal Transduct Target Therapy.

[2] Hoogmartens J, Cacace R, Van Broeckhoven C (2021). Insight into the genetic etiology of Alzheimer’s Disease: a comprehensive review of the role of rare variants. Alzheimer’s & Dementia: Diagnosis Assessment & Disease Monitoring.

[3] Hampel H, Hardy J, Blennow K et al. The Amyloid-β pathway in Alzheimer’s Disease. Mol Psychiatry. 2021: 1–23. 10.1038/s41380-021-01249-0 PMC8758495 34456336

[4] Sturchio A, Dwivedi AK, Young CB (2021). High cerebrospinal amyloid-β 42 is associated with normal cognition in individuals with brain amyloidosis. EClinicalMedicine.

[5] Storck SE, Hartz AM, Bernard J (2018). The concerted amyloid-beta clearance of LRP1 and ABCB1/P-gp across the blood-brain barrier is linked by PICALM. Brain Behav Immun.

[6] Deane R, Du Yan S, Submamaryan RK (2003). RAGE mediates amyloid-β peptide transport across the blood-brain barrier and accumulation in brain. Nat Med.

[7] Storck SE, Meister S, Nahrath J (2016). Endothelial LRP1 transports amyloid-β 1–42 across the blood-brain barrier. J Clin Investig.

[8] Pflanzner T, Janko MC, André-Dohmen B (2011). LRP1 mediates bidirectional transcytosis of amyloid-β across the blood-brain barrier. Neurobiol Aging.

[9] Yamada K, Hashimoto T, Yabuki C (2008). The low density lipoprotein receptor-related protein 1 mediates uptake of amyloid β peptides in an in vitro model of the blood-brain barrier cells. J Biol Chem.

[10] Bierhaus A, Humpert PM, Morcos M (2005). Understanding RAGE, the receptor for advanced glycation end products. J Mol Med.

[11] Roher AE, Esh CL, Kokjohn TA (2009). Amyloid beta peptides in human plasma and tissues and their significance for Alzheimer’s Disease. Alzheimer’s Dement.

[12] Nag S, Begley D. Blood-brain barrier, exchange of metabolites and gases. Pathology and Genetics Cerebrovascular Diseases. 22 – 9; 2005.

[13] Sweeney MD, Kisler K, Montagne A, Toga AW, Zlokovic BV (2018). The role of brain vasculature in neurodegenerative disorders. Nat Neurosci.

[14] Sweeney MD, Sagare AP, Zlokovic BV (2018). Blood–brain barrier breakdown in Alzheimer Disease and other neurodegenerative disorders. Nat Reviews Neurol.

[15] Chan Y, Chen W, Wan W, Chen Y, Li Y, Zhang C (2018). Aβ1–42 oligomer induces alteration of tight junction scaffold proteins via RAGE-mediated autophagy in bEnd. 3 cells. Exp Cell Res.

[16] Kook S-Y, Hong HS, Moon M, Ha CM, Chang S, Mook-Jung I (2012). Aβ1–42-RAGE interaction disrupts tight junctions of the blood–brain barrier via Ca2+-calcineurin signaling. J Neurosci.

[17] Wan W, Cao L, Liu L (2015). Aβ1–42 oligomer-induced leakage in an in vitro blood–brain barrier model is associated with up‐regulation of RAGE and metalloproteinases, and down‐regulation of tight junction scaffold proteins. J Neurochem.

[18] Van Dorpe J, Smeijers L, Dewachter I (2000). Prominent cerebral amyloid angiopathy in transgenic mice overexpressing the London mutant of human APP in neurons. Am J Pathol.

[19] Weller RO, Massey A, Newman TA, Hutchings M, Kuo Y-M, Roher AE (1998). Cerebral amyloid angiopathy: amyloid β accumulates in putative interstitial fluid drainage pathways in Alzheimer’s Disease. Am J Pathol.

[20] Zhang L, Chen C, Mak MS (2020). Advance of sporadic Alzheimer’s Disease animal models. Med Res Rev.

[21] Hsiao K, Chapman P, Nilsen S (1996). Correlative memory deficits, Aβ elevation, and amyloid plaques in transgenic mice. Science.

[22] Kumar-Singh S, Pirici D, McGowan E (2005). Dense-core plaques in Tg2576 and PSAPP mouse models of Alzheimer’s Disease are centered on vessel walls. Am J Pathol.

[23] Callahan MJ, Lipinski WJ, Bian F, Durham RA, Pack A, Walker LC (2001). Augmented senile plaque load in aged female β-amyloid precursor protein-transgenic mice. Am J Pathol.

[24] Drummond E, Wisniewski T (2017). Alzheimer’s Disease: experimental models and reality. Acta Neuropathol.

[25] Hanafy AS, Steinlein P, Pitsch J (2023). Subcellular analysis of blood-brain barrier function by micro-impalement of vessels in acute brain slices. Nat Commun.

[26] McIlwain H (1958). Maintenance of the composition of isolated cerebral tissues. Neurology.

[27] Schindelin J, Arganda-Carreras I, Frise E (2012). Fiji: an open-source platform for biological-image analysis. Nat Methods.

[28] Joshi P, Turola E, Ruiz A (2014). Microglia convert aggregated amyloid-β into neurotoxic forms through the shedding of microvesicles. Cell Death & Differentiation.

[29] Irudayanathan FJ, Trasatti JP, Karande P, Nangia S (2016). Molecular architecture of the blood brain barrier tight junction proteins-A synergistic computational and in vitro approach. J Phys Chem B.

[30] Essodaigui M, Broxterman H, Garnier-Suillerot A (1998). Kinetic analysis of Calcein and Calcein – acetoxymethylester efflux mediated by the Multidrug Resistance Protein and P-Glycoprotein. Biochemistry.

[31] Hubensack M, Müller C, Höcherl P (2008). Effect of the ABCB1 modulators elacridar and tariquidar on the distribution of paclitaxel in nude mice. J Cancer Res Clin Oncol.

[32] Holló Z, Homolya L, Hegedüs T, Sarkadi B (1996). Transport properties of the multidrug resistance-associated protein (MRP) in human tumour cells. FEBS Lett.

[33] Deane R, Singh I, Sagare AP (2012). A multimodal RAGE-specific inhibitor reduces amyloid β–mediated brain disorder in a mouse model of Alzheimer Disease. J Clin Investig.

[34] Brunk UT, Terman A (2002). Lipofuscin: mechanisms of age-related accumulation and influence on cell function. Free Radic Biol Med.

[35] Ujiie M, Dickstein DL, Carlow DA, Jefferies WA (2003). Blood–brain barrier permeability precedes senile plaque formation in an Alzheimer Disease model. Microcirculation.

[36] Poduslo JF, Curran GL, Wengenack TM, Malester B, Duff K (2001). Permeability of proteins at the blood–brain barrier in the normal adult mouse and double transgenic mouse model of Alzheimer’s Disease. Neurobiol Dis.

[37] Bourasset F, Ouellet M, Tremblay C (2009). Reduction of the cerebrovascular volume in a transgenic mouse model of Alzheimer’s Disease. Neuropharmacology.

[38] Knox EG, Aburto MR, Clarke G, Cryan JF, O’Driscoll CM (2022). The blood-brain barrier in aging and neurodegeneration. Mol Psychiatry.

[39] Löscher W, Friedman A (2020). Structural, molecular, and functional alterations of the blood-brain barrier during epileptogenesis and Epilepsy: a cause, consequence, or both?. Int J Mol Sci.

[40] Lam FC, Liu R, Lu P (2001). β-Amyloid efflux mediated by p‐glycoprotein. J Neurochem.

[41] Vogelgesang S, Cascorbi I, Schroeder E (2002). Deposition of Alzheimer’s β-amyloid is inversely correlated with P-glycoprotein expression in the brains of elderly non-demented humans. Pharmacogenet Genomics.

[42] Hartz AM, Miller DS, Bauer B (2010). Restoring blood-brain barrier P-glycoprotein reduces brain amyloid-β in a mouse model of Alzheimer’s Disease. Mol Pharmacol.

[43] Krohn M, Lange C, Hofrichter J (2011). Cerebral amyloid-β proteostasis is regulated by the membrane transport protein ABCC1 in mice. J Clin Investig.

[44] Miller MC, Tavares R, Johanson CE (2008). Hippocampal RAGE immunoreactivity in early and advanced Alzheimer’s Disease. Brain Res.

[45] LaRue B, Hogg E, Sagare A (2004). Method for measurement of the blood–brain barrier permeability in the perfused mouse brain: application to amyloid-β peptide in wild type and Alzheimer’s Tg2576 mice. J Neurosci Methods.

[46] Carrano A, Hoozemans JJ, Van Der Vies SM, Van Horssen J, De Vries HE, Rozemuller AJ (2012). Neuroinflammation and blood-brain barrier changes in capillary amyloid angiopathy. Neurodegenerative Dis.

[47] Scheltens P, De Strooper B, Kivipelto M (2021). Alzheimer’s Disease. The Lancet.

[48] FDA Grants. Accelerated approval for Alzheimer’s drug. FDA; 2021.

[49] FDA Grants. Accelerated approval for Alzheimer’s Disease Treatment. FDA; 2023.

[50] Sevigny J, Chiao P, Bussière T (2016). The antibody aducanumab reduces Aβ plaques in Alzheimer’s Disease. Nature.

[51] Van Dyck CH, Swanson CJ, Aisen P (2023). Lecanemab in early Alzheimer’s Disease. N Engl J Med.

[52] Zhang S, Zhi Y, Li F (2018). Transplantation of in vitro cultured endothelial progenitor cells repairs the blood-brain barrier and improves cognitive function of APP/PS1 transgenic AD mice. J Neurol Sci.

[53] Keaney J, Walsh DM, O’Malley T (2015). Autoregulated paracellular clearance of amyloid-β across the blood-brain barrier. Sci Adv.

[54] Greene C, Kealy J, Humphries M (2018). Dose-dependent expression of claudin-5 is a modifying factor in schizophrenia. Mol Psychiatry.

[55] Burstein A, Sabbagh M, Andrews R, Valcarce C, Dunn I, Altstiel L (2018). Development of Azeliragon, an oral small molecule antagonist of the receptor for advanced glycation endproducts, for the potential slowing of loss of cognition in mild Alzheimer’s Disease. J Prev Alzheimer’s Disease.

