# Supplementary material for: Local perfusion of capillaries reveals disrupted beta-amyloid homeostasis at the blood-brain barrier in Tg2576 murine Alzheimer’s model
Source: Fluids Barriers CNS. 2023 Nov 22;20:85. doi: 10.1186/s12987-023-00492-7 (PMC10666337; doi:10.1186/s12987-023-00492-7)
Supplement: Supplementary file 1 — Supplementary Material 1 [file 12987_2023_492_MOESM1_ESM.docx]

**Supplementary information**

The detailed ANOVA table and pairwise comparisons in **Fig. 3e**, generated by GraphPad Prism.

|  | **SS** | **DF** | **MS** | **F (DFn, DFd)** | **P value** |
| --- | --- | --- | --- | --- | --- |
| **Interaction** | 4388 | 3 | 1463 | F (3, 32) = 2.807 | *P=0.0553* |
| **Row Factor** (blockers) | 261415 | 3 | 87138 | F (3, 32) = 167.3 | *P<0.0001* |
| **Column Factor** (genotype) | 31248 | 1 | 31248 | F (1, 32) = 59.98 | *P<0.0001* |
| **Residual** | 16671 | 32 | 521.0 |  |  |

| **Tukey's multiple comparisons test** | **Adjusted P Value** |
| --- | --- |
| WT No blockers vs. WT PRO | 0.0778 |
| WT PRO vs. WT ELA | 0.0001 |
| WT ELA vs. WT PRO+ELA | <0.0001 |
| Tg2576 No blockers vs. Tg2576 PRO | 0.9555 |
| Tg2576 PRO vs. Tg2576 ELA | <0.0001 |
| Tg2576 ELA vs. Tg2576 PRO+ELA | <0.0001 |
| WT No blockers vs. Tg2576 No blockers | 0.0106 |

The detailed ANOVA table and pairwise comparisons in **Fig. 4b**, generated by GraphPad Prism.

| **Capillaries** | **SS** | **DF** | **MS** | **F (DFn, DFd)** | **P value** |
| --- | --- | --- | --- | --- | --- |
| **Interaction** | 7508 | 1 | 7508 | F (1, 39) = 13.27 | *0.0008* |
| **Row Factor** (blocker) | 7326 | 1 | 7326 | F (1, 39) = 12.95 | *0.0009* |
| **Column Factor** (genotype) | 22017 | 1 | 22017 | F (1, 39) = 38.92 | *<0.0001* |
| **Residual** | 22061 | 39 | 565.7 |  | |

| **Tukey's multiple comparisons test** | **Adjusted P Value** |
| --- | --- |
| WT No blockers vs. WT FPS-ZM1 | <0.0001 |
| Tg2576 No blocker vs. Tg2576 FPS-ZM1 | >0.9999 |
| WT No blockers vs. Tg2576 No blockers | <0.0001 |

The detailed ANOVA table and pairwise comparisons in **Fig. 4d**, generated by GraphPad Prism.

| **Venules** | **SS** | **DF** | **MS** | **F (DFn, DFd)** | **P value** |
| --- | --- | --- | --- | --- | --- |
| **Interaction** | 90303 | 1 | 90303 | F (1, 49) = 5.038 | *0.0293* |
| **Row Factor** (blocker) | 81470 | 1 | 81470 | F (1, 49) = 4.546 | *0.0380* |
| **Column Factor** (genotype) | 23333 | 1 | 23333 | F (1, 49) = 1.302 | *0.2594* |
| **Residual** | 878228 | 49 | 17923 |  |  |

| **Tukey's multiple comparisons test** | **Adjusted P Value** |
| --- | --- |
| WT No blockers vs. WT FPS-ZM1 | 0.0415 |
| Tg2576 No blocker vs. Tg2576 FPS-ZM1 | 0.9997 |
| WT No blockers vs. Tg2576 No blockers | 0.0394 |
